# 

**Published:** 1872-02

**Authors:** 


☞ Should any of our subscribers or exchanges fail to get THE COMPANION regularly, they will confer a favor by notifying us of the fact. Our Mailing Clerk will faithfully forward THE COMPANION. Subscribers will please notify Post masters that it will reach their offices, if not lost on the way, between the 15th and 20th of every month. Should it fail to come, after a reasonable time, notify the Editors and it will be sent.

☞ We respectfully solicit Original Articles, Reports of Societies, Clinics Home and Foreign Correspondence, News, etc., etc., of interest to medical readers.

☞ To insure publication, articles should be practical, as brief as possible, and carefully written, on one side of the paper. Articles should be received by the 15th of one month to insure publication in the next. ☞ Friends, send in your articles. ☜

☞ All subscribers and others will please write their names, post-offices, county and State *plainly*

### SUBSCRIBERS, TAKE NOTICE.

We ask our subscribers again to *do us* the favor to notify their post-masters that The Companion will, unless lost on the way, reach their offices between the 15th and 20th of each month. When it fails to come, notify the editors. We desire, especially, that our friends may have a complete set of volume number two, commencing with the January number 1872. Hence, we request them to notify us *at once* if they have not yet received it, as the edition is being rapidly exhausted.

We are gratified to state that our subscription-list is being increased rapidly; as almost every medical gentleman seeing the practical character of The Companion, becomes a subscriber at once. The success of The Georgia Medical Companion is unprecedented in the history of medical journals of the South. From the very start, it established itself permanently in the esteem of the true professional men of the country, and is now, by their kindness, and love of principle, the largest circulated medical journal of its age, on the continent.

We wish to make it the best known and most widely circulated monthy in the country, and therefore, ask our friends, everywhere, to forward us the names of their medical friends whom they think would like to subscribe for a journal of its character.

